# Artemether-lumefantrine efficacy among adults on antiretroviral therapy in Malawi

**DOI:** 10.1186/s12936-023-04466-w

**Published:** 2023-01-27

**Authors:** Wongani Nyangulu, Randy G. Mungwira, Titus H. Divala, Nginache Nampota-Nkomba, Osward M. Nyirenda, Andrea G. Buchwald, Jernelle Miller, Dominique E. Earland, Matthew Adams, Christopher V. Plowe, Terrie E. Taylor, Jane E. Mallewa, Joep J. van Oosterhout, Sunil Parikh, Matthew B. Laurens, Miriam K. Laufer, the TSCQ Study Team

**Affiliations:** 1 Dignitas International, Zomba, Malawi; 2 Blantyre Malaria Project, Kamuzu University of Health Sciences, Blantyre, Malawi; 3 Center for Vaccine Development and Global Health, University of Maryland School of Medicine, 685 W. Baltimore St., Baltimore, MD 21201 USA; 4 Michigan State University, East Lansing, USA; 5 Department of Medicine, Kamuzu University of Health Sciences, Blantyre, Malawi; 6 Yale School of Public Health, New Haven, USA; 7 Present Address: Public Health and Nutrition Research Group, Kamuzu University of Health Sciences, Mangochi, Malawi; 8 Present Address: Partners in Hope, Lilongwe Malawi and David Geffen School of Medicine, University of California, Los Angeles, USA

## Abstract

**Background:**

When people with human immunodeficiency virus (HIV) infection (PWH) develop malaria, they are at risk of poor anti-malarial treatment efficacy resulting from impairment in the immune response and/or drug-drug interactions that alter anti-malarial metabolism. The therapeutic efficacy of artemether-lumefantrine was evaluated in a cohort of PWH on antiretroviral therapy (ART) and included measurement of day 7 lumefantrine levels in a subset to evaluate for associations between lumefantrine exposure and treatment response.

**Methods:**

Adults living with HIV (≥ 18 years), on ART for ≥ 6 months with undetectable HIV RNA viral load and CD4 count ≥ 250/mm^3^ were randomized to daily trimethoprim-sulfamethoxazole (TS), weekly chloroquine (CQ) or no prophylaxis. After diagnosis of uncomplicated *Plasmodium falciparum* malaria, a therapeutic efficacy monitoring was conducted with PCR-correction according to WHO guidelines. The plasma lumefantrine levels on day 7 in 100 episodes of uncomplicated malaria was measured. A frailty proportional hazards model with random effects models to account for clustering examined the relationship between participant characteristics and malaria treatment failure within 28 days. Pearson’s Chi—squared test was used to compare lumefantrine concentrations among patients with treatment failure and adequate clinical and parasitological response (ACPR).

**Results:**

411 malaria episodes were observed among 186 participants over 5 years. The unadjusted ACPR rate was 81% (95% CI 77–86). However, after PCR correction to exclude new infections, ACPR rate was 94% (95% CI 92–97). Increasing age and living in Ndirande were associated with decreased hazard of treatment failure. In this population of adults with HIV on ART, 54% (51/94) had levels below a previously defined optimal day 7 lumefantrine level of 200 ng/ml. This occurred more commonly among participants who were receiving an efavirenz-based ART compared to other ART regimens (OR 5.09 [95% CI 1.52–7.9]). Participants who experienced treatment failure had lower day 7 median lumefantrine levels (91 ng/ml [95% CI 48–231]) than participants who experienced ACPR (190 ng/ml [95% CI 101–378], p-value < 0.008).

**Conclusion:**

Recurrent malaria infections are frequent in this population of PWH on ART. The PCR-adjusted efficacy of AL meets the WHO criteria for acceptable treatment efficacy. Nevertheless, lumefantrine levels tend to be low in this population, particularly in those on efavirenz-based regimens, with lower concentrations associated with more frequent malaria infections following treatment. These results highlight the importance of understanding drug-drug interactions when diseases commonly co-occur.

## Background

HIV and malaria infections are endemic in sub-Saharan Africa, and previous research demonstrated high rates of clinical malaria illness among adults living with HIV infection after stopping trimethoprim-sulfamethoxazole prophylaxis [1]. Thus, evaluating anti-malarial treatment efficacy in this population is critical. Artemether–lumefantrine (AL) is the most common artemisinin-based combination therapy in sub–Saharan Africa and is the first-line anti-malarial treatment of uncomplicated malaria in Malawi [2]. The World Health Organization (WHO) recommends that all first-line anti-malarial medicines in a national treatment policy have treatment success ≥ 90% as assessed by monitoring therapeutic efficacy [3]. People with HIV infection (PWH) (may be at risk of experiencing higher rates of anti-malarial drug failure than the general population because of anti-malarial drug resistance, impaired immunity, increased malaria parasite biomass and/or sub-therapeutic anti-malarial drug levels. To distinguish treatment failure secondary to anti-malarial drug resistance from that caused by sub-therapeutic anti-malarial drug levels, pharmacokinetic and therapeutic efficacy studies are required.

Non-nucleoside reverse transcriptase inhibitors have been the most common antiretrovirals used in sub-Saharan Africa since the roll-out of antiretroviral therapy (ART)on the continent. These drugs are extensively metabolized by cytochrome p450 (CYP450) enzymes and thus interact with compounds that use similar pathways. In particular, efavirenz induces CYP 3A4 and CYP 2B6, which metabolize lumefantrine into desbutyl-lumefantrine, leading to reduced plasma levels of lumefantrine and increased risk of malaria treatment failure [4–7].While concentrations of lumefantrine on day 7 ranging from 175 ng/mL to 280 ng/mL have been shown to ensure adequate therapeutic response to AL in HIV infected and uninfected children and adults, a recent meta-analysis suggested a minimum day 7 lumefantrine concentration of 200 ng/ml is required to ensure adequate clinical response to AL after treatment for uncomplicated malaria [8].

High rates of malaria illness in PWH who discontinued trimethoprim-sulfamethoxazole (TS) or chloroquine (CQ) prophylaxis were reported from this randomized clinical trial, evaluating the role of anti-malarial prophylaxis among PWH on antiretroviral therapy in Malawi [1]. Participants with thick blood smear-confirmed uncomplicated malaria were enrolled in a 28-day therapeutic efficacy study, and a sub-group submitted samples for day 7 lumefantrine levels. The objectives were to determine the therapeutic efficacy of AL among PWH and to distinguish treatment failure from inadequate drug levels.

## Methods

The study design and methodology for the parent clinical trial have been published previously [9]. In summary, PWH over 18 years of age on antiretroviral therapy were recruited into a randomized open—label phase III clinical trial. Participants were randomized in a 1:1:1 ratio to (1) continue standard of care of daily TS prophylaxis (160 mg trimethoprim/ 800 mg sulfamethoxazole), (2) discontinue TS prophylaxis and begin weekly CQ prophylaxis (300–310 mg chloroquine base), or (3) discontinue TS prophylaxis. Participants were followed up every 4–12 weeks, and whenever they were ill.

Study participants were recruited from two sites in Malawi: Ndirande research clinic in Blantyre, an urban centre, and Tisungane ART clinic at Zomba Central Hospital, located in a more rural setting. Of note, the two sites have different malaria burdens: malaria parasite prevalence in children under 5 years old is 4% in Ndirande versus 28% in Zomba [10]. Potential participants underwent informed consent before any study-related procedures. At screening, all participants had complete medical history, full physical examination, and blood samples collected for complete blood count, alanine aminotransferase, creatinine, CD4 count and HIV viral load. Consenting adults were recruited if they had: (1) been on ART for at least 6 months, (2) undetectable HIV viral load of < 400 copies/mm^3^, and (3) CD4 count of at least 250/mm^3^. Additional inclusion/exclusion criteria are published with the protocol [9].

### Malaria diagnosis and species differentiation

Participants who presented with malaria symptoms were evaluated at the study clinic and had blood collected for microscopy. Uncomplicated malaria was defined as objective fever (temperature ≥ 37.5 °C) or history of fever within the past 48 h and/or other symptoms such as nausea, vomiting and diarrhoea, headache, back pain, chills, or myalgia, plus detection of any malaria parasites in blood by microscopy. Participants with danger signs such as reduction or loss of consciousness and difficulty breathing were referred to the hospital and not enrolled in the therapeutic efficacy study. Two trained microscopists identified and quantified malaria parasitaemia from thick blood smears and determined *Plasmodium* species using thin blood smears. Only infections with *P. falciparum* are included here.

### Malaria treatment and follow up

Participants diagnosed with uncomplicated *P. falciparum* malaria were treated with 80 mg artemether and 480 mg lumefantrine twice daily for 3 days. First doses were directly observed. All participants were followed up on days 1, 2, 3, 7, 14, 21 and 28 according to WHO standard for monitoring therapeutic efficacy, and blood smears and dried blood spots were collected at each visit.

In cases of recurrent malaria infection occurring on or after day 14, dried blood spots collected on filter paper from enrolment day and the day of recurrence of infection underwent extraction to distinguish new from recrudescent infection by genotyping merozoite surface protein-1 (MSP-1), MSP-2 and the glutamate-rich protein (GLURP) according to the publicly available protocol [11].

Participants who consented to participate in a sub-study of lumefantrine drug concentration measurements were selected to submit blood specimens for day 7 drug levels. Participants were only enrolled 1 time. These participants were given 250 ml of milk to drink with each dose to ensure optimal and consistent lumefantrine absorption [12]. Doses 1, 3 and 5 of anti-malarial treatment were administered under direct observation. On day 7 of follow up for monitoring therapeutic efficacy, 5 ml of blood was collected and centrifuged at 3000 rpm for 10 min to collect 2 ml of plasma. Immediately after centrifugation, plasma was stored at – 20 °C. Once a week, plasma samples were transferred to a – 80 °C freezer for storage at the central laboratory.

Plasma was shipped to the Parikh laboratory at the Yale School of Public Health (New Haven, CT) on dry ice, and immediately transferred to NorthEast BioLab (Hamden, CT) for drug level analysis of lumefantrine and desbutyl-lumefantrine using liquid chromatography-tandem mass spectrometry on an API 5000 triple-quadruple mass spectrometer (Applied Biosystems/MDS SCIEX, Foster City, CA). The calibration range was 10.9–3785 ng/mL for lumefantrine, and 1.9–1130 ng/mL for desbutyl-lumefantrine with the lower limit of quantification (LLOQ) at 10.0 and 1.9 ng/mL for lumefantrine and desbutyl-lumefantrine, respectively. The coefficient of variation was 1.4% and 5.6% for lumefantrine and desbutyl-lumefantrine, respectively.

### Definitions

*Treatment failure* was diagnosed according to WHO criteria which classify outcomes in mutually exclusive groups [13].

*Early treatment failure* was defined as any of the following: danger signs or severe malaria on day 1, 2 or 3 in the presence of parasitaemia; parasitaemia on day 2 higher than on day 0, irrespective of axillary temperature; parasitaemia on day 3 with axillary temperature ≥ 37.5 °C; parasitaemia on day 3 ≥ 25% of count on day 0.

*Late clinical failure* was defined as any of the following: danger signs, severe malaria, or axillary temperature ≥ 37.5 °C in the presence of parasitaemia on any day between day 4 and day 28 in patients who did not previously meet early treatment failure criteria.

*Late parasitological failure* was defined as any of the following: presence of parasitaemia on any day between day 7 and day 28 with axillary temperature < 37.5 °C in patients who did not previously meet early treatment failure or late clinical failure criteria.

*Adequate clinical and parasitological response* (ACPR) was defined as: absence of parasitaemia on days 28, irrespective of axillary temperature, in patients who did not previously meet early treatment failure, late clinical failure or late parasitological failure criteria.

After analysis of genotyping results, an infection was recrudescent if any genotype identified in the recurrent infection was also present at the initial infection. In PCR-adjusted results, only recrudescent infections were considered treatment failures.

### Statistical analysis

Kaplan Meier survival analysis was used to determine the cumulative success rate defined as not reaching a failure point during the time under observation. Individuals diagnosed with malaria who did not have a 28 day follow-up visit and did not meet treatment failure definitions were censored at their last visit. was estimated to assess for possible predictors of treatment failure, including prophylactic regimen, age, sex, ART regimen, presentation with fever, parasite density at presentation, and study site. A random effect for individual was included to account for multiple observations among the same individuals. For the analysis of prophylactic regimen, the CQ and TS treatment arms were combined due to few malaria episodes among individuals on prophylaxis.

To examine the association between lumefantrine and desbutyl-lumefantrine concentrations and treatment failure, a Wilcoxon Rank Sum test was used to compare the distribution of lumefantrine concentration between patients with treatment failure and those with ACPR. Because the majority of desbutyl-lumefantrine levels were below the level of detection, these levels were classified as detectable or undetectable. Not all participants with concentration data had complete follow-up data. Due to the limited number of data points, participants who were followed up to at least 14 days without treatment failure were classified as treatment successes, participants who were not followed at either 14, 21, or 28 days were excluded. As a day 7 lumefantrine concentration of 200 ng/ml has been associated with higher likelihood of treatment success, the analysis examined whether this cut-off point was associated with treatment failure in this cohort using Pearson’s Chi—squared test. All statistical analysis was conducted in SAS version 9.4 (Cary, NC, USA).

### Ethical considerations

The study was approved by the Kamuzu University of Health Sciences Research Ethics Committee and the University of Maryland Baltimore Institutional Review Board. All participants provided written informed consent.

## Results

From December 2012 to July 2018, clinical outcomes for 411 clinical malaria episodes among 186 participants (Table 1) were detected and measured. The geometric mean parasite density on the day malaria was diagnosed for each episode was 3394 (95% CI 2828, 4073)/microlitre. Eight episodes were classified as early clinical failure, 35 episodes were late clinical failure, and 22 episodes were late parasitological treatment failure. Fifty episodes with incomplete follow up were censored at the time of their last visit. Among 57 episodes with recurrent parasitaemia between 14 and 28 days, 15 episodes did not have samples available for genotyping and were censored at 14 days for the PCR-adjusted analysis.

**Table 1:** Demographic characteristics

	Overall	Ndirande	Zomba
Number of participants	186	97	89
Study arm			
CQ, N(%)	16 (8.6)	11 (11.3)	5 (5.6)
TS, N(%)	25 (13.4)	15 (15.5)	10 (11.2)
No prophylaxis, N(%)	145 (78.0)	71 (73.2)	74 (83.2)
Follow up time, days, median (IQR)	924 (756, 1,596)	1,596 (1,315, 1,833)	756 (686, 840)
Age (years), mean (SD)	42.0 (9.9)	40.3 (9.6)	43.7 (9.9)
Sex			
Female, N(%)	147 (79.0)	78 (80.4)	69 (77.5)
BMI (kg/m^2^)			
Underweight (< 18.5)	15 (8.1)	6 (6.2)	9 (10.1)
Healthy weight (18.5–24.9)	142 (76.3)	69 (71.1)	73 (82.0)
Overweight (25–29.9)	22 (11.8)	16 (16.5)	6 (6.7)
Obese (30 or greater)	7 (3.8)	6 (6.2)	1 (1.1)
ART regimen (7 missing)			
With EFV, N(%)	142 (79.3)	70 (72.2)	72 (87.8)
Non EFV, N(%)	37 (20.7)	27 (27.8)	10 (12.2)
Bed net use			
Never, N(%)	51 (27.4)	26 (26.8)	25(28.1)
Last night, N(%)	133 (71.5)	69 (71.1)	64 (71.9)
Number of Clinical Malaria Episodes, mean (range)	2.4 (1, 14)	1.4 (1, 5)	3.5 (1, 14)
Parasite density on day 0 of malaria episode, geometric mean (95% CI)	3394 (2828, 4073)	3278 (2322, 4629)	3450 (2783, 4278)

In unadjusted results, 283/346 treated malaria episodes followed until day 28 resulted in ACPR, treatment efficacy of 81% (95% CI 77–86). After PCR correction, the cumulative efficacy was 94% (95% CI 92, 97). The hazard of failure was higher among participants who received CQ or TS prophylaxis compared to those who did not (although, few participants in this analysis were on prophylaxis), men compared to women, and participants who received efavirenz vs other ART regimens, though none of these differences achieved statistical significance (Table 2). Of note, participants in the Ndirande site were less likely to experience treatment failure compared to their counterparts in Zomba (HR 0.21, 95% CI 0.07–0.60).

**Table 2:** Distribution of episodes with adequate response, censored episodes, and treatment failure by study arm, ART regimen, and other risk factors for failure

	Adequate response	Censored	ECF	Late failure (LFC/LPF)	Any failure (uncorrected)	PCR corrected failure/total included	Uncorrected cumulative survival rate (95% CI)^a^	PCR corrected cumulative survival rate (95% CI)^a^	Failure HR (95% CI)^b^
Overall (n = 411)	283	65	8	57	65	20/396	0.81 (0.77, 0.86)	0.94 (0.92, 0.97)	–
Study treatment arm									
No prophylaxis (n = 341)	243	51	3	48	51	12/327	0.83 (0.78, 0.87)	0.96 (0.94, 0.98)	1.00 (REF)
CQ or TS (n = 70)	40	14	5	9	14	8/69	0.76 (0.65, 0.87)	0.87 (0.79, 0.96)	1.56 (0.76, 3.2)
Sex									
Female (n = 323)	222	49	3	46	49	13/309	0.82 (0.77, 0.86)	0.95 (0.93, 0.98)	1.00 (REF)
Male (n = 88)	61	16	5	11	16	7/87	0.80 (0.71, 0.89)	0.92 (0.86, 0.98)	1.34 (0.67, 2.70)
ART treatment regimen									
On EFV-based ART (n = 332)	229	57	8	49	57	19/322	0.80 (0.76, 0.85)	0.94 (0.91, 0.96)	1.56 (0.67, 3.66)
Other ART regimens (n = 79)	54	8	0	8	8	1/74	0.87 (0.79, 0.95)	0.98 (0.95, 1.02)	1.00 (REF)
Fever at day 0									
No Fever (n = 311)	217	53	8	45	53	19/302	0.81 (0.76, 0.85)	0.93 (0.9, 0.96)	1.00 (REF)
Fever (n = 100)	66	12	0	12	12	1/94	0.84 (0.76, 0.92)	0.99 (0.96, 1.01)	0.70 (0.35, 1.38)
Parasite density									
Parasite density, mean (SD)^c^	11344 (16865)	11677 (17243)	1995 (2410)	13167 (18060)	11677 (17243)	9235.8 (22289.6)	–	–	1.03 (0.89, 1.19)
Age									
Age, mean (SD)^d^	43.6 (9.9)	40.5 (8.3)	38.3 (7.7)	40.8 (8.4)	40.5 (8.3)	40.5 (8.7)	–	–	0.97 (0.94, 1.00)
Study site									
Ndirande (n = 130)	77	4	0	4	4	0/127	0.95 (0.9, 1)	1.00 (N/A)	0.21 (0.07, 0.60)
Zomba (n = 281)	206	61	8	53	61	20/269	0.77 (0.72, 0.82)	0.92 (0.89, 0.96)	1.00 (REF)

*LPF* late parasitological failure, *LCF* late clinical failure, *ECF* early clinical failure

^a^Cumulative survival rate and 95% CI calculated from Kaplan Meier survival analysis

^b^Hazard ratios from frailty proportional hazards model, including random effect for individual to account for multiple observations per individual. Failure HR shown for models with total PCR uncorrected failure rates. Models with PCR corrected failure rates showed consistent results but had insufficient numbers of events for a valid analysis

^c^Parasite density log-transformed for hazard ratio calculation, estimate is for one log increase in parasite density

^d^Estimate for age indicates the HR for a 1 year increase in age

Day seven plasma lumefantrine levels were collected in 100 participants. Six were excluded from analysis due to lack of follow-up data. One individual had a value below the LLOQ for lumefantrine and the sample was assigned a value half the LLOQ. The median day 7 lumefantrine level in the remaining 94 participants was 186 ng/ml (IQR 95,359). A statistically significant difference was observed between participants who experienced treatment failure vs. ACPR (91 ng/ml (IQR 48–231) vs 190 ng/ml (IQR 101–378), p-value < 0.008) (Table 3).

**Table 3:** Lumefantrine concentration by treatment outcome among 94 individuals

Outcome	Number of observations	Mean lumefantrine at day 7 (SD)	Median lumefantrine at day 7 (IQR)	Lumefantrine level below 200 ng/ml, N(%)	OR for lumefantrine below 200 ng/ml (95% CI)
Total	94	247 (201)	186 (95, 359)	51 (54.3)	
Treatment outcome**					
Failure	20	143 (108)	91 (48, 231)	13 (65.0)	1.76 (0.63, 4.91)
Success	74	270 (208)	190 (101, 378)	38 (51.4)	Reference
ART regimens***					
Efavirenz	77	197 (144)	170 (86, 277)	47 (61.0)	5.09 (1.52, 17.09)
Non-efavirenz	17	448 (270)	383 (296, 666)	4 (23.5)	Reference

Desbutyl lumefantrine results		N detectable at day 7 (%)	N undetectable at day 7 (%)	OR for undetectable desbutyl lumefantrine (95% CI)
Treatment outcome				
Failure	20	7 (35.0)	13 (65.0)	3.05 (1.09, 8.56)
Success	74	46 (62.2)	28 (37.8)	Reference
ART regimens				
Efavirenz	77	48 (62.3)	29 (37.7)	0.25 (0.08, 0.79)
Non-efavirenz	17	5 (29.4)	12 (70.6)	Reference

^**^p < 0.01 by Wilcoxon rank sum test

^***^p < 0.001 by Wilcoxon rank sum test

Drug levels were low in this population with 54.3% of individuals having a day 7 lumefantrine concentrations less than 200 ng/ml. Among those with treatment failure, 65.0% had an undetectable desbutyl-lumefantrine level (Table 3). Participants on efavirenz-based ART regimens were significantly more likely to have lumefantrine concentrations below 200 ng/ml (OR = 5.09, 95% CI = 1.52, 17.09) and this ART regimen was also aartssociated with a lower likelihood of having an undetectable desbutyl-lumefantrine level (OR = 0.25 95%CI = 0.08, 0.79). Among the 94 individuals followed up to at least 14 days, treatment failure was consistently associated with low lumefantrine and desbutyl-lumefantrine levels. However, there was no statistically significant difference in treatment failure comparing those with lumefantrine concentrations below 200 ng/ml to those above (OR = 1.76, 95% CI = 0.63, 4.91).

## Discussion

In this evaluation of AL therapeutic efficacy among PWH who are well controlled on ART, unadjusted overall treatment efficacy was lower than expected at 81% but after PCR-correction to exclude new infections, the adjusted treatment efficacy rate was 94%. This meets the WHO requirement for first-line anti-malarial agents to have at least 90% efficacy in therapeutic efficacy studies. This also confirms that the observed high rate of recurrent infection is not likely due to drug resistance. Although these findings confirm that resistance to AL is not clinically detectable in Malawi, high rates of new infections may be due to low lumefantrine levels that fail to protect against subsequent infections.

Although the 94% efficacy is an acceptable rate, the results are lower than 99% treatment efficacy documented in a population of children around the same time in Malawi [14]. This may suggest that the decreased lumefantrine levels associated with efavirenz interaction have a modest impact of AL efficacy. In a previous publication from this site, HIV-associated immunosuppression did not impact antimalarial treatment efficacy [15]. Of note, the treatment efficacy among PWH on efavirenz-based ART was documented as 100% in neighbouring Zambia [16], but that study documented an initial geometric mean parasite density of 1108 parasites/microlitre, compared to 3394 in this study and may explain the difference in efficacy.

The day seven 7 plasma lumefantrine levels were low in our population of adults living with HIV Recurrent parasitaemia was associated with lower lumefantrine levels. The low drug concentrations and recurrent parasitaemia were both more common in participants on efavirenz-based ART regimen than those taking other regimens, consistent with results of a previous meta-analysis [7]. In that analysis of drug interactions between lumefantrine and commonly used ART regimens, lumefantrine exposure was significantly decreased (up to 47% lower) with efavirenz based ART compared to other ART regimens, leading to frequent sub therapeutic drug concentrations.

This study also supports the induction effect of efavirenz on lumefantrine metabolism through quantification of its primary metabolite, desbutyl-lumefantrine [17]. Efavirenz use was associated with higher levels of the desbutyl metabolite, which was hypothesized to reflect the efavirenz-based induction of CYP3A4-mediated lumefantrine metabolism as compared to those on non-efavirenz-based regimens. While most of the study participants achieved ACPR with malaria treatment, other studies in PWH have demonstrated reduction in therapeutic efficacy when Efavirenz based ART was co-administered with lumefantrine. In a Ugandan study, PWH on an efavirenz-based regimen had day 28 ACPR of 82.5% when treated with AL [18].

A higher rate of treatment failure was observed among participants living in Zomba, a more rural area, compared to Blantyre, an urban centre. Malaria transmission intensity is higher in Zomba compared with Blantyre. Therefore, participants in Zomba were exposed to more infectious bites of *Anopheles* mosquitoes which increases the risk of treatment failure due to reinfection, though that was excluded by PCR correction. In addition, with higher density infections and more polyclonal infections, the PCR assay has more sensitivity to detect a low frequency recurrent clone. This explanation is supported by models that suggest false positive identification of recrudescent infections occur in higher transmission settings [19].

Although the study did not identify a concerning level of anti-malarial resistance as evidenced by the 94% efficacy rate, low drug levels have the potential to impact the emergence and spread of resistance [20]. Sub-therapeutic drug levels provide an environment that allow drug resistant parasites to survive treatment and/or allow for resistant newly infecting clones to outcompete more sensitive clones. Fortunately, most PWH also receive TS prophylaxis, which prevents most clinical cases of malaria [1]. Thus, the reservoir of parasites exposed to low lumefantrine levels in this population will be small. However, if TS prophylaxis is discontinued, these drug interactions could have significant public health impact on resistance emergence and spread.

## Conclusion

The PCR-corrected therapeutic efficacy of AL among PWH in this setting was within the required range, but patients on an efavirenz-based regimen experienced lower lumefantrine levels than patients on other ART highlighting the importance of drug-drug interactions. Drug-drug interactions for common infections can alter pharmacokinetics and pharmacodynamics and have significant impact on individual and public health outcomes. Although efavirenz is largely being replaced by dolutegravir in sub-Saharan Africa, it will remain in use in a smaller population. In addition, this report highlights the need to consider common infections and associated pharmacokinetic interactions in the wider drug development and drug policy decisions.

## Data Availability

Data are available from the corresponding author on request.

## Abbreviations

ACPR: Adequate clinical and parasitological response
AL: Artemether-lumefantrine
ART: Antiretroviral therapy
CI: Confidence interval
CQ: Chloroquine
GLURP: The glutamate-rich protein
LLOQ: Lower limit of quantification
MSP: The glutamate-rich protein
OR: Odds ratio
PWH: People with HIV
TS: Trimethoprim sulfamethoxazole
WHO: World Health Organization
